# Identification of inorganic compounds in composite alum-treated wooden artefacts from the Oseberg collection

**DOI:** 10.1038/s41598-018-21314-z

**Published:** 2018-02-13

**Authors:** Caitlin M. A. McQueen, Diego Tamburini, Susan Braovac

**Affiliations:** 1 Museum of Cultural History, University of Oslo, Postboks 6762 St. Olavs plass, 0130 Oslo, Norway; 2 Department of Chemistry and Industrial Chemistry, University of Pisa, via Moruzzi 13, I-56124 Pisa, Italy; 3 Present Address: Department of Scientific Research, The British Museum, Great Russell Street, London, WC1B 3DG United Kingdom

## Abstract

Alum-treated wooden artefacts from the Oseberg collection display a great deal of morphological, structural and compositional inhomogeneity. Thus, an in-depth understanding of chemical processes underlying their degradation requires consideration of a variety of local environments. In addition to alum, sources of inorganic compounds include metal parts, corrosion products of which can migrate into the surrounding wood. In order to characterise the inorganic compounds a range of local environments, samples from several locations in a selection of composite objects have been investigated by X-ray diffraction (XRD), Fourier transform infrared (FTIR) spectroscopy, Raman spectroscopy and scanning electron microscopy (SEM)-energy dispersive X-ray spectroscopy (EDS). We have found that corrosion of iron rods used in reconstruction has formed iron(II) sulfates, which have migrated into the alum-treated wood to form sulfates containing combinations of potassium, aluminium, iron(II) and iron(III) cations. Reactions of alum were also evident from the presence of alunite in some samples. Areas with significant abundances of zinc sulfates, zinc sulfide and elemental sulfur were also detected. These results provide a first-time window into the complex array of inorganic species that can be present in such composite alum-treated objects.

## Introduction

Artefacts from the Oseberg burial, housed at the Viking Ship Museum in Oslo, Norway, represent one of the most comprehensive collections of Viking Age wooden objects in the world. Upon excavation in the early 1900s, hot (ca. 90 °C) concentrated solutions of alum (KAl(SO_4_)_2_·12H_2_O) were used to conserve the more deteriorated waterlogged wood. Objects were subsequently treated and restored using a range of materials including nails and screws, glues, putties, linseed oil and varnishes^1–3^. A substantial amount of these objects is therefore made up of non-wood components, including various inorganic compounds.

We know now that alum treatment was ultimately very damaging to the wood, believed to be largely due to the ensuing release of sulfuric acid^4^. However, elemental analyses have also revealed a complex mixture of inorganic elements in the wood, arising from conservation treatment, metal corrosion and incidental migration. Analyses of organic components have highlighted correlations between wood degradation (loss of carbohydrates and lignin oxidation) and the presence of some of these elements, thus suggesting that these may also play a role in the decay. The presence of iron ions in particular could be an accelerating factor^3,5,6^ due to their catalytic role in wood degradation^7,8^.

The detrimental effect of iron ions on archaeological wooden objects has been the subject of several studies^9–14^. However, the previous work on iron in archaeological wood has focussed on artefacts from marine environments, in which the combination of corroding iron and products from sulfate reducing bacteria had resulted in accumulation of iron sulfides that began to oxidise after excavation. The Oseberg collection, however, was found buried in a terrestrial site, and while the artefacts also contain a lot of sulfur, it is mainly in the form of sulfates introduced during the alum treatment^6^. Furthermore, most of the literature studies involve wood treated with polyethylene glycol (PEG), which has been widely used to conserve waterlogged wood since the 1960s^15^. Although the alum method was a common treatment for artefacts excavated prior to this, especially in Scandinavia, research on the chemistry of alum-treated wood is much less abundant^3–6,16^. However, the need for improved chemical understanding of this material is increasing rapidly as the consequences of alum-treatment become more evident. Thus, the investigation into the Oseberg collection is an important case study with potentially wide-ranging implications.

We have previously described facets of this work focussing on the chemical characterisation of highly degraded alum-treated wood fragments^4–6^. However, these fragments were only treated with alum, and therefore do not represent many objects in the collection that have been restored with additional materials. We herein describe sampling and analysis of a range of typical local environments found in the more complex Oseberg artefacts, with a focus on inorganic components such as corroding iron parts, in order to gain insight into specific alteration/degradation pathways in a wider range of objects.

## Materials and Methods

### Samples

**Figure 1:**
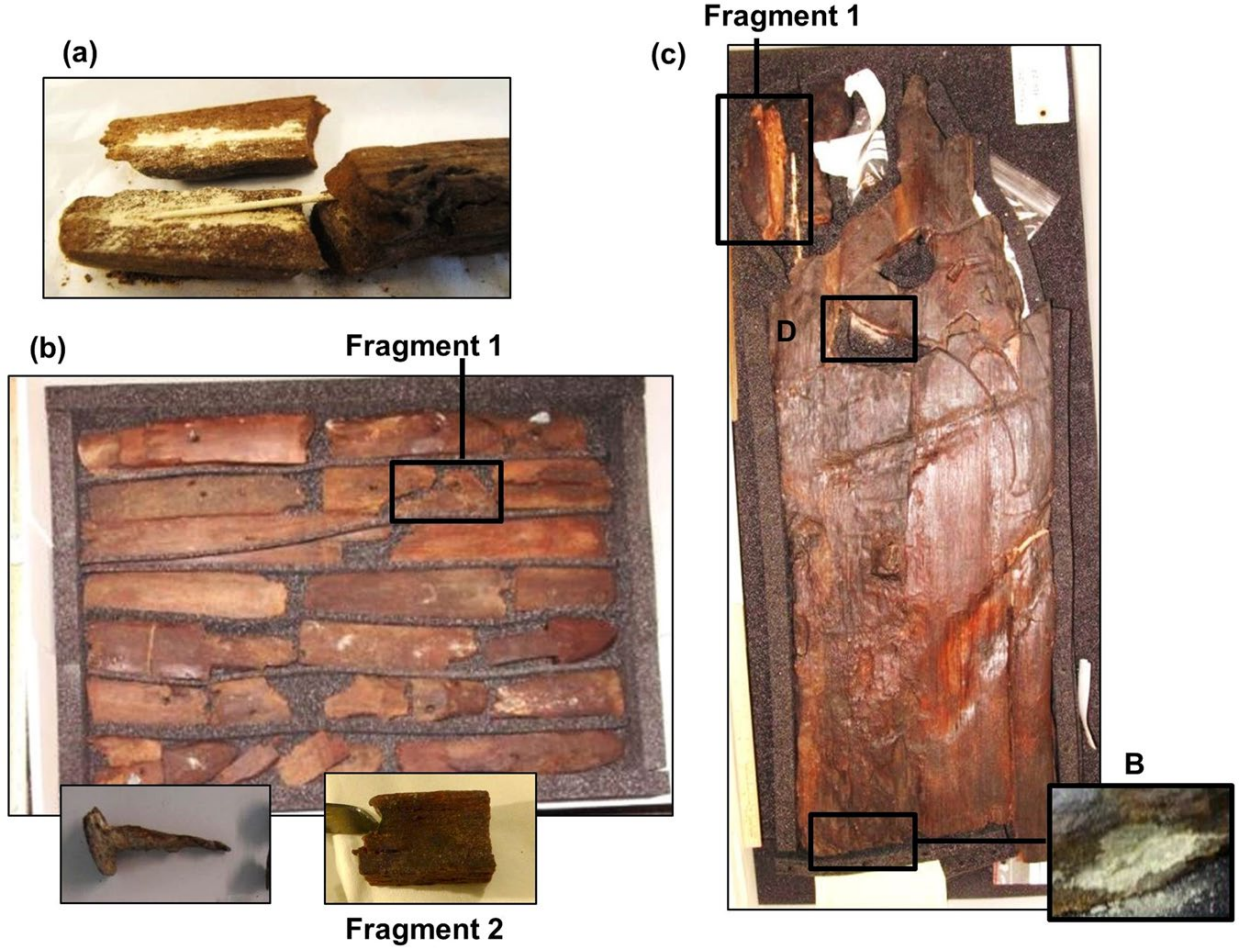
Objects and fragments from which samples were taken: (**a**) fragment from object 195; (**b**) wooden pieces and nail from object 210, including fragments 1 and 2; (**c**) object 250 showing fragment 1, off-white efflorescence B and brown efflorescence D.

The samples come from various regions of three separate objects containing corroded iron parts, and are summarised in Table 1. Three samples (195A, 195B and 195C) were taken from an uncoated alum-treated fragment from object 195, a sled. This fragment contains an iron rod that was introduced during reconstruction (Fig. 1a). Some inorganic analyses of the samples from this fragment have been previously described in a preliminary study^17^.

**Table 1:** Description of the samples analysed.

Sample name	Description
195A	Off-white powder from surface of exposed iron rod
195B	Wood from inner part of fragment underneath off-white powder
195C	Wood from inner part of fragment, outer edge
210A	Corrosion product from original nail
210B	Linseed oil coated wood next to an original nail remnant, fragment 1
210C	Linseed oil coated wood inside hole without remnant nail, fragment 2
210D	Linseed oil coated wood from end of fragment 2 without hole or nail
210E	Linseed oil impregnated wood from inside fragment 2
250B	Off-white efflorescence on linseed oil coated wood surface
250C	Off-white powder from surface of exposed iron rod
250D	Brown efflorescence from linseed oil coated wood surface
250E	Wood from inner part of fragment 1 adjacent to iron rod
250F	Wood from inner part of fragment 1, between rod adjacent area and coated surface
250G	Wood from inner part of fragment 1, linseed oil coated outer edge

Object 210 consists of pieces of an unreconstructed barrel, wooden parts of which have been treated with alum and coated with linseed oil (Fig. 1b). Original nails or pieces thereof remain in some fragments, and some are stored separately. One sample (210A) was taken from the corrosion layer of one of these nails. Another sample (210B) was taken from a wood fragment 1, next to an original nail remnant. Three samples (210C, 210D and 210E) were taken from different parts of a separate wood fragment 2.

Object 250 is a carved wooden board, thought to have been part of a bed post, which has been treated with alum, reconstructed with iron rods and coated with linseed oil and varnish (Fig. 1c). Two samples (250B and 250D) were taken from separate areas of efflorescence on the wood surface. One sample (250C) was taken from the corrosion layer on the surface of an exposed metal rod. Three samples (250E, 250F and 250G) were taken from different parts of a wood fragment 1 adjacent to the exposed rod.

A szomolnokite (FeSO_4_∙H_2_O) sample, kindly provided by the Natural History Museum, Oslo, was used as a reference for FTIR and Raman spectroscopy.

### X-ray imaging

X-ray images were acquired using the Museum of Cultural History’s in-house X-ray setup consisting of a Comet MR 225 KV X-ray tube with an ISOVOLT Titan E generator (45 mA) and control module. Images were acquired using industrial phosphor imaging plates (IPS), placed inside CRxFlex Rigid Cassettes with a 0.250 mm Pb front. Imaging plates were processed using a CRxFLEX Scanner, using the Rhythm RT 5.0 acquistion software. Images were viewed using the Rhythm Review software (Radiography Release 4.3). The image was captured at settings 90 kV, 3 mA and 100 seconds.

### FTIR spectroscopy

FTIR spectra in ATR mode were recorded on a Thermo Scientific Nicolet iS50 spectrometer equipped with a diamond crystal and DTGS detector. Spectra were recorded with 32 scans at 4 cm^−1^ resolution, within the range 4000–400 cm^−1^.

Micro-infrared (μ-FTIR) spectroscopy was performed using synchrotron radiation (SR) at the IRIS beamline, BESSY II synchrotron facility, Helmholtz-Zentrum Berlin, Germany. A Nicolet Nexus 870 spectrometer and Nicolet Continuμm FTIR microscope was used to perform transmission experiments using 32× magnification with apertures between 10–20 microns. 128 scans were recorded per spectrum at a spectral resolution of 4 cm^−1^, within the range 4000–800 cm^−1^.

Spectra were graphed using Origin 2017 (OriginLab, Northampton, MA).

### Raman microscopy

Raman spectra were recorded with a Renishaw inVia Raman miscroscope, using a 488 nm laser at 100% power. Laser power, exposure time and the number of accumulations were modified depending on the sample. The reference spectra of sulfur and krausite were obtained from the RRUFF Project database (RRUFF ID R040135 and R110211, respectively)^18^.

Spectra were graphed using Origin 2017 (OriginLab, Northampton, MA).

### XRD

X-ray diffraction analysis was carried out using a PANalytical diffractometer Empyrean Series 2 with radiation CuKα1 = 1.54 Å, operating at 45 kV, 40 mA, 2θ range 8–70°, step size 0.03°, time per step 5000s, equipped with a PIXcel^1D^-Medipix3 RTMS detector, and High Score data acquisition and interpretation software. A zero background sample holder was used. Crystalline phases were identified using the ICDD database.

### SEM-EDS

Analyses were performed using a FEI Quanta 450 Scanning Electron Microscope coupled with an Oxford X-Max^N^ 50mm^2^ detector, using low vacuum mode to avoid charging and a voltage of 20 kV. The other parameters (spot size, pressure, and working distance) were modified depending on the sample.

### Data availability

The datasets generated during the current study are available from the corresponding author on reasonable request.

## Results and Discussion

### Iron corrosion

**Figure 2:**
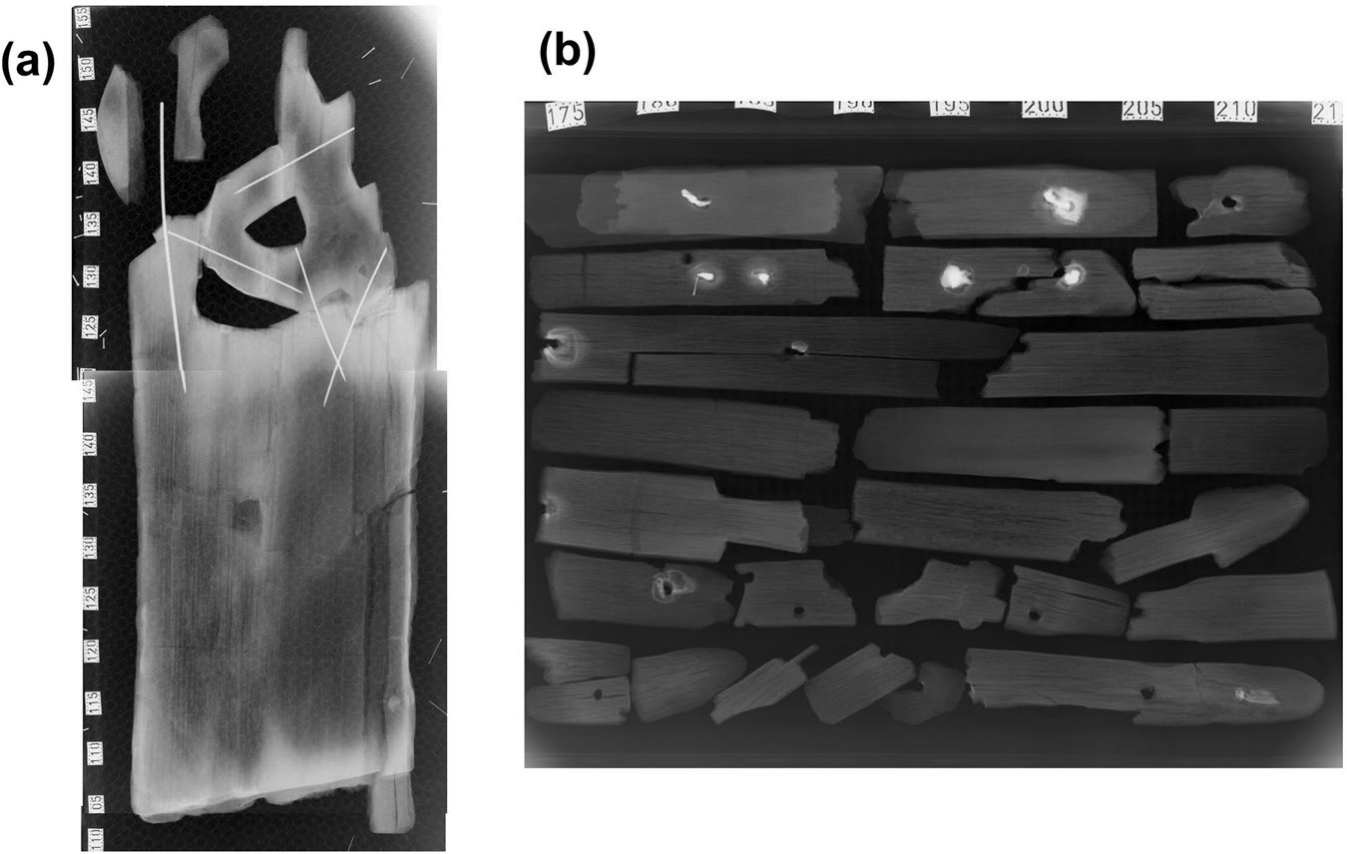
X-ray images of (**a**) object 250, showing metal rods (bright white lines) from reconstruction; (**b**) fragments of object 210, some with remnant nails, seen as bright white areas in the image.

The metal rods in objects 195 and 250, confirmed as being made of iron by SEM-EDS, were introduced during reconstruction in the early 1900s. The internal rods in object 250 are visible in the X-ray image shown in Fig. 2a.

**Figure 3:**
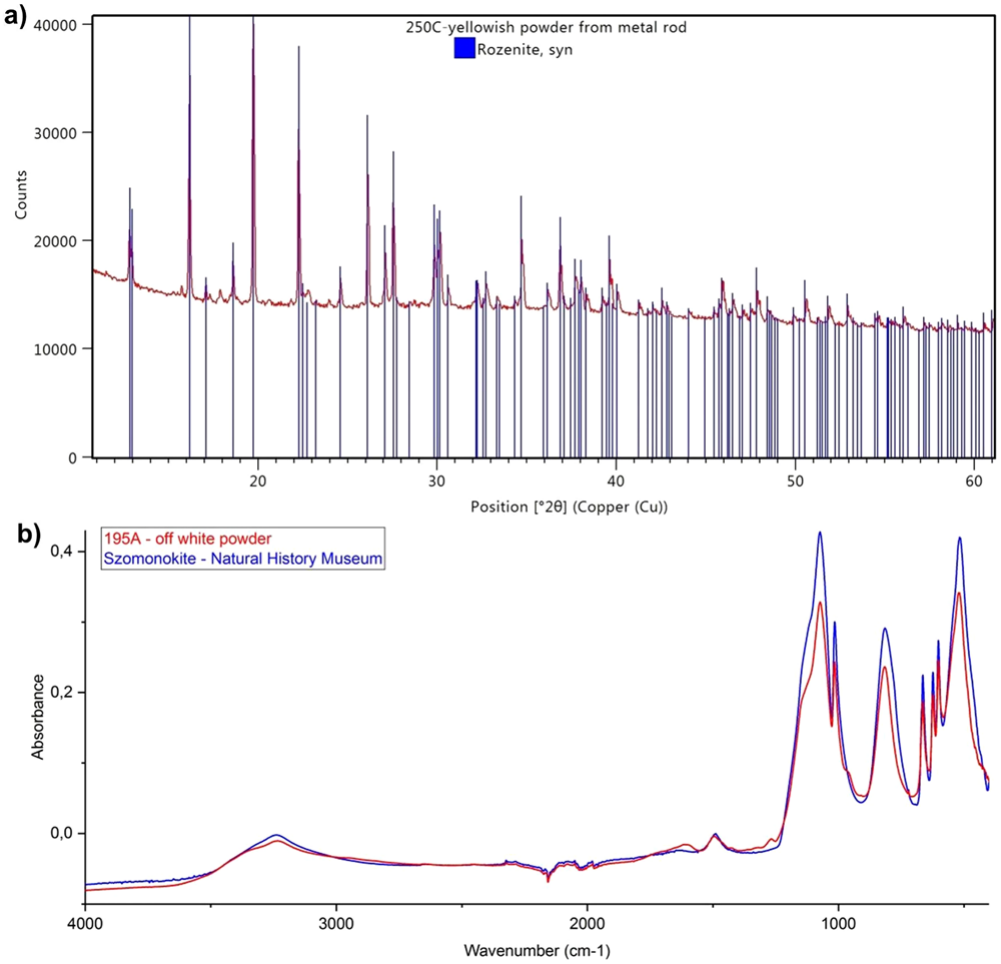
FeSO_4_ formed on the surface of iron rods in alum-treated wood, as seen in (**a**) the X-ray diffraction pattern from sample 250C (shown with rozenite reference); (**b**) the ATR-FTIR spectrum of sample 195A (shown with szomolnokite reference).

**Figure 4:**
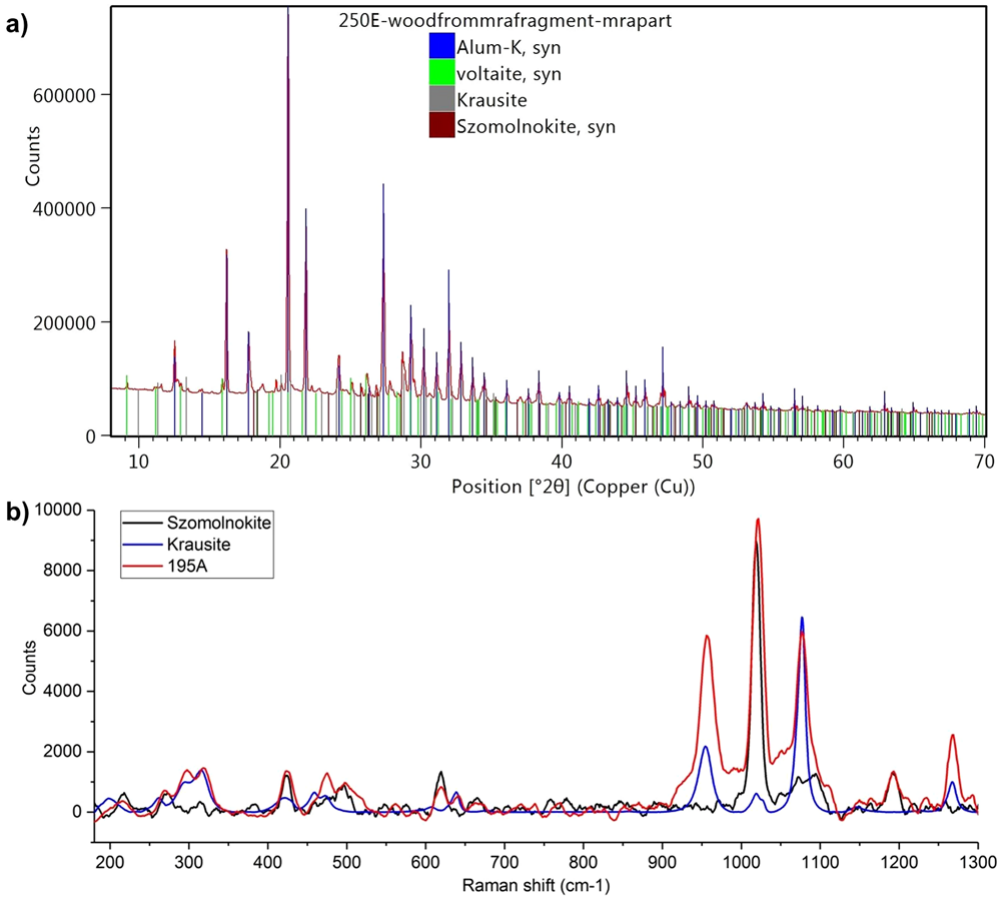
Products of reactions between alum and iron species as seen in (**a**) X-ray diffraction pattern from sample 250E (shown with reference patterns of alum, voltaite, krausite and szomolnokite); (**b**) the Raman spectrum of sample 195A (shown with szomolnokite and krausite references).

A thick layer of powdery off-white corrosion product was observed on the surfaces of the exposed rods. This was unsurprising, given that the wood is known to be highly acidic (pH strips indicated pH ≤2 in these fragments), which would accelerate oxidation of iron. Samples of these powders, 195A and 250C, were found to mainly consist of szomolnokite (FeSO_4_∙H_2_O) and rozenite (FeSO_4_∙4H_2_O), respectively, by powder XRD (Fig. 3a)^17^. This was supported by infrared spectroscopy (Fig. 3b). These soluble minerals could provide a readily available source of ferrous ions for oxidative degradation of the wood. XRD also detected szomolnokite in wood samples 195B, 195C and 250E taken from near these corroding iron pieces (Fig. 4a)^17^.

Other iron-containing minerals were also detected in the powders (195A and 250C) and nearby wood samples (195B,C and 250E). Potassium iron(III) sulfate was detected in the diffraction patterns of samples 250E, 195A and 195B as the monohydrate krausite (KFe(SO_4_)_2_∙H_2_O), supported by Raman spectroscopy (Fig. 4b). The tetrahydrate form goldichite (KFe(SO_4_)_2_∙4H_2_O) was identified in samples 195B and 195C. Additionally, XRD suggested the presence of the mineral voltaite (K_2_Fe^2+^_5_Fe^3+^_3_Al(SO_4_)_12_(H_2_O)_18_), containing mixed oxidation states of iron (Fig. 4a). These compounds demonstrate further iron oxidation and reactions with alum (or its decomposition products) as the corrosion products migrate into the wood.

A pale yellowish efflorescence, 250B, was also observed on the surface of object 250 at the opposite end from the exposed metal rod, yet was found to contain rozenite as a significant component by XRD (see XRD pattern of 250B in *Other inorganic compounds*). This was surprising, as the X-ray image (Fig. 2a) shows that there are no metal rods close to this area. This might therefore be a result of earlier storage of the object in contact with an iron part.

**Figure 5:**
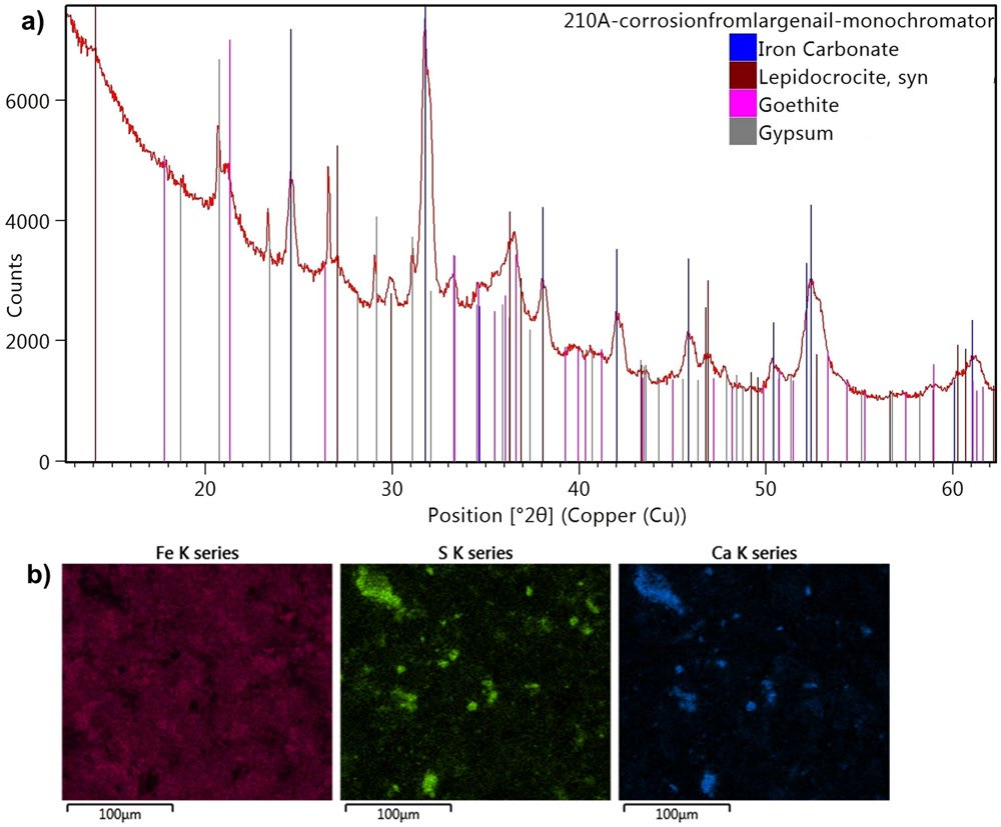
(**a**) X-ray diffraction pattern from sample 210A, with reference patterns for iron carbonate, lepidocrocite, goethite and gypsum; (**b**) SEM-EDS elemental maps of Fe, S and Ca from sample 210A showing S-rich areas mainly corresponding to Ca-rich areas.

In contrast, corrosion products on an original nail from object 210 (sample 210A) were found to include iron carbonate and FeO(OH) (lepidocrocite and goethite) by XRD (Fig. 5a), FTIR and Raman spectroscopy. No iron sulfates could be detected, and although sulfur was observed by SEM-EDS, S-rich areas mainly overlapped with Ca-rich areas (Fig. 5b). This is consistent with the presence of some gypsum (CaSO_4_∙2H_2_O) in the corrosion layer, also observed in the XRD pattern. This common mineral occurs naturally in Norway^19^ and was likely deposited during burial.

Absence of iron sulfates could indicate that the nails were removed from the wood prior to alum treatment. However, as visible in Fig. 2b, pieces of nails remained in some of the alum-treated wood fragments and sample 210B, taken from wood adjacent to a nail remnant, did not have detectable amounts of iron sulfates either. Since nails from this object are made from iron produced during the Viking age, and were extensively corroded prior to contact with alum, we cannot directly compare them as materials to the iron rods introduced during reconstruction. Furthermore, the linseed oil coating may provide a protective barrier between the nail and the acidic wood. Unfortunately, it was not possible to obtain analogous samples from objects with either linseed oil coating and recently introduced iron parts, or no coating and original iron parts.

**Figure 6:**
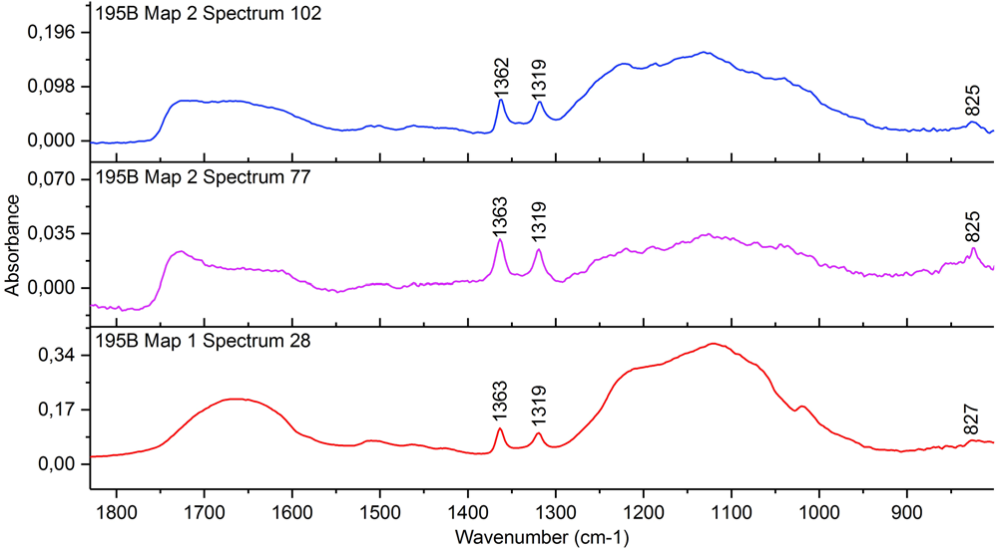
SR μ-FTIR spectra from sample 195B showing peaks around 1363, 1319 and 825 cm^−1^ that may correspond to humboldtine^20^.

Apart from sulfates, carbonates and oxyhydroxides, one other iron compound was potentially identified. In sample 195B, recurring peaks in several μ-FTIR spectra around 1363, 1319 and 825 cm^−1^ did not correspond to wood, alum or any of the iron sulfates detected by XRD (Fig. 6). These are consistent with literature values for infrared bands of the iron oxalate mineral humboldtine (FeC_2_O_4_∙2H_2_O)^20^. However, all these spectra contained additional absorptions from various other compounds in the sample, and some regions of distinctive bands of humboldtine were obscured. Additionally, some minor peaks in the XRD pattern of sample 210B matched those of the humboldtine reference, but again the presence of other compounds prevented unambiguous identification of this compound. The suggested presence of iron oxalate is nonetheless interesting, given that we have previously discussed the possibility that iron oxalate species could play a role in deterioration mechanisms of wood from the Oseberg collection^5^.

### The state of alum

**Figure 7:**
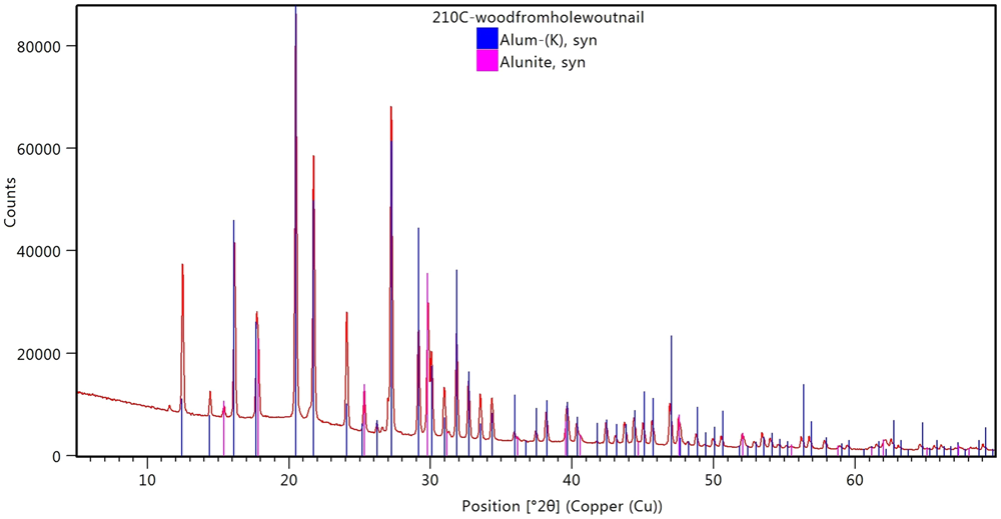
X-ray diffraction pattern from 210C showing alum and alunite.

In addition to the products of reactions with iron, alum transformation was observed by the presence of alunite, KAl_3_(SO_4_)_2_(OH)_6_, in the XRD patterns of all surface wood samples (B-D) from object 210 (Fig. 7). This hydroxide is known to form due to hydrolysis in hot aqueous solutions of alum, as shown in Equation (1)^3,4^. Alunite was not detected in sample 210E from the inner region of one of the fragments, suggesting that it settled on the surface of wood fragments during treatment, rather than forming from a slow reaction of alum in the wood.

1 $$3\,{\rm{KAl}}{({{\rm{SO}}}_{4})}_{2}+12\,{{\rm{H}}}_{2}{\rm{O}}\to {{\rm{KAl}}}_{3}{({{\rm{SO}}}_{4})}_{2}{({\rm{OH}})}_{6}+2\,{{\rm{K}}}^{+}+4\,{{{\rm{SO}}}_{4}}^{2-}+6\,{{\rm{H}}}_{3}{{\rm{O}}}^{+}$$

We recently noted the recurring presence of mercallite (KHSO_4_) in other alum-treated wood samples from the Oseberg collection^6^. However, mercallite was not detected in any of the samples in the present study. Nor was arcanite (K_2_SO_4_) detected, which may form for lower sulfuric acid concentrations than those that favour mercallite^21^. Although potassium and sulfate ions may have preferentially formed compounds with iron, such as krausite, near the iron rods, neither mercallite nor arcanite was observed even in those samples containing no detectable potassium iron sulfates (250F-G and 210B-E). Given that the previous samples were treated only with alum, while the present samples contain various additional materials and are a lot more variable in composition, it is difficult to suggest an explanation for the absence of mercallite based on the results of these few samples, but is an issue that could be looked into further.

### Other inorganic compounds

**Figure 8:**
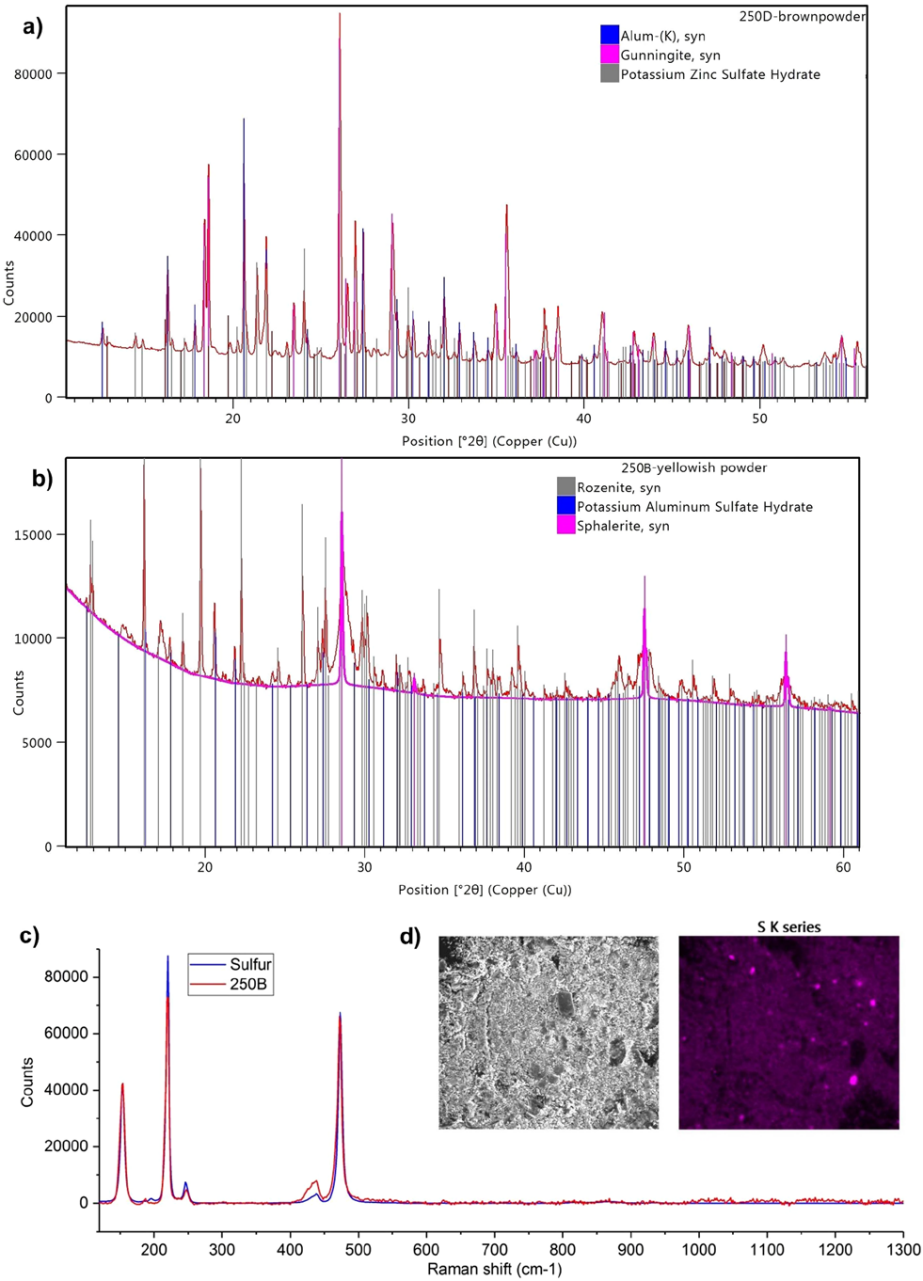
X-ray diffraction patterns and spectra showing zinc compounds elemental sulfur in efflorescence samples from object 250: (**a**) XRD pattern 250D with alum, gunningite and K_2_(Zn(H_2_O)_6_)(SO_4_)_2_ references; (**b**) XRD pattern 250B with reference patterns for rozenite, alum and sphalerite (highlighted); (**c**) Raman spectrum of sulfur from 250B; (**d**) SEM-EDS elemental map of S from 250D.

Efflorescence was observed and sampled in two areas of object 250. These were both found to contain significant amounts of zinc compounds. Significant levels of zinc were observed in other wood samples from the Oseberg collection^5^, and were attributed to zinc tanks that were used to store the objects after excavation. Sample 250D, from a brown efflorescence, contained significant amounts of ZnSO_4_∙H_2_O (gunningite) and K_2_(Zn(H_2_O)_6_)(SO_4_)_2_ (Fig. 8a). The latter compound was supported by SEM-EDS phase analysis, and was also identified in the X-ray diffraction pattern of wood sample 195C^17^.

A third zinc compound was detected in the XRD pattern from the off-white efflorescence 250B, which showed peaks consistent with ZnS (sphalerite) (Fig. 8b). In addition, elemental sulfur was detected in Raman spectra of both efflorescence samples (Fig. 8c). This was supported by SEM-EDS, which showed spots of concentrated S abundance that did not correspond with any other elements (Fig. 8d). It was somewhat unexpected to find these sulfur species, as the oxidised sulfate component of alum is believed to be the main source of this element. Though accumulation of reduced sulfur species is well documented in the wood of marine archaeological shipwrecks due to sulfate-reducing bacteria^10,22–25^, they have not been associated with archaeological wood from terrestrial sites to our knowledge. However, Rosenqvist previously noted the presence of native sulfur in the Oseberg burial mound, and that clays like those at the burial site have pore water with sulfate levels comparable to sea water^2^. This, combined with the low oxygen environment of the burial, would provide a good environment for sulfate-reducing bacteria^26^.

## Conclusions

XRD, SEM-EDS, FTIR spectroscopy and Raman spectroscopy have been performed on samples from alum-treated composite artefacts from the Oseberg collection in order to identify inorganic compounds present in various local environments. The analyses have revealed that accelerated corrosion of iron rods in the acidic wood has resulted in the formation of iron(II) sulfates on their surface, causing conservation concerns due to the readily accessible ferrous ions. These have migrated into the wood to a certain extent and reacted further with alum (or degradation products thereof) to form sulfates containing combinations of potassium, aluminium, iron(II) and iron(III) cations. However, iron sulfates did not appear to have formed to any appreciable degree in alum and linseed oil treated wood surrounding an original iron nail. It remains unclear whether this is due to the linseed oil coating or to the original nail remnant being less prone to corrosion than the more recently introduced rods.

Alunite was also identified as a by-product of alum decomposition on the surface of some wood pieces, presumably formed during treatment of the objects at 90 °C. KHSO_4_, which has been previously identified in wood from Oseberg artefacts, was not present in detectable amounts in any of these samples. Further investigations into samples from composite objects will hopefully provide better insight into this latter observation.

Additionally, various zinc compounds and sulfur in reduced forms (relative to sulfate) were detected in some samples. The former presumably results from storage of the objects in zinc tanks post-excavation, while the latter supports a previous suggestion that sulfate-reducing bacteria were present in the Oseberg burial environment.

Thus, these results have revealed a complex array of inorganic compounds present in the composite artefacts, which are incredibly variable between local environments, even within the same object. This information is in agreement with the high variability in chemical composition previously observed for other alum-treated wood samples from the Oseberg collection. Further work is planned to investigate whether the presence of inorganic compounds is related to the state of preservation of the wood. This improved understanding of the chemical nature of composite alum-treated artefacts will also be valuable when considering suitable preservation strategies.
